# Resin Infiltration of Non-Cavitated Enamel Lesions in Paediatric Dentistry: A Narrative Review

**DOI:** 10.3390/children9121893

**Published:** 2022-12-02

**Authors:** Nabihah Dziaruddin, Ahmad Shuhud Irfani Zakaria

**Affiliations:** 1 Department of Paediatric Dentistry and Orthodontics, Faculty of Dentistry, Universiti Malaya, Kuala Lumpur 50603, Malaysia; 2 Department of Family Oral Health, Faculty of Dentistry, The National University of Malaysia, Kuala Lumpur 50300, Malaysia

**Keywords:** resin infiltration, paediatric dentistry, minimal intervention dentistry, dental caries, dental developmental anomalies

## Abstract

The resin infiltration (RI) technique was introduced as one of the minimal intervention dentistry strategies in addressing dental caries among the paediatric population. This technique used the low-viscosity resin monomer to infiltrate the non-cavitated carious lesion and other developmental enamel porosities, thus allowing the conservation of the tooth structure. This narrative review aims to explore the value of RI in Paediatric Dentistry. Through our search of the literature, the development of the material, their clinical applications and shortcomings, as well as the innovation that has been carried out to improve the current RI, were discussed. There are number of high-level evidence supporting the use of RI in arresting non-cavitated proximal caries lesions in primary and permanent teeth, but its efficacy in managing anterior white spot lesions is still unclear. Limited penetration depth, not radiopaque and questionable long-term colour and material stability were among the limitation of the material. Various laboratory-based studies have been conducted to improve the current properties of RI. Nevertheless, RI has emerged as one of the important micro-invasive techniques in addressing non-cavitated and anterior white-spot enamel lesions in children and adolescents with great success.

## 1. Introduction

Managing dental caries among the paediatric population is a never-ending story. Although dental caries is preventable, it has affected more than 600 million children worldwide [1], and without prompt intervention, the disease can progress and increase the morbidity of the child [2]. With a better understanding of the etiological factors and behaviour of dental caries, the concept of caries management in the modern era has now changed [3]. The minimal intervention dentistry (MID) concept has been introduced, emphasising early detection of dental caries and the promotion of preventive therapy. Advancement of the lesion should be managed by adopting the minimally invasive operative approach [4]. Thanking Buonocore [5] and Bowen [6] for their discovery of the acid etching technique and the bisphenol A-glycidyl methacrylate (Bis-GMA) resin monomer, respectively, various resin-based dental materials that support the MID concept have been developed.

Resin infiltrant (RI), marketed as Icon^®^ (DMG, Hamburg, Germany), is among the products that benefited from Buonocore’s and Bowen’s breakthrough discovery. The RI technique has emerged as one of the important tools that support the MID concept in managing non-cavitated carious lesions, using the tagline ‘Drilling? No thanks!’. This novel material uses the concept of infiltrating the caries lesion using a low-viscosity resin in an attempt to arrest the progression of the non-cavitated caries lesion without the need of removing the tooth structure. This concept is highly desirable and well appreciated, especially in the field of Paediatric Dentistry, since the drilling procedure may not be well-tolerated by young and highly anxious children [7]. 

This narrative review article aims at exploring the usage of the RI technique in Paediatric Dentistry. We divided the paper into three sections: the past, where we revisited the early development of the RI; the present, where we discussed the current utilisation of the techniques in paediatric patients and their shortcomings and the future, where the innovation and improvisation that has been made to the current RI formulation were explored.

## 2. The Past: Development of RI

### 2.1. The Resin Monomer

Surprisingly, the idea of infiltrating early caries lesions has existed for more than five decades. Researchers since the 1970s have conducted many in vitro and in vivo studies to find the right material that is able to infiltrate the early enamel lesion effectively [8,9,10,11,12,13]. 

Going back to 1975, Davila and colleagues [8] investigated the potential ability of adhesives to seal early enamel lesions. They proposed an idea that the demineralised surface of the lesion would act as a suitable locus for the adhesives to infiltrate, sealing the lesion entrance and spaces, thus arresting the ongoing demineralisation and preventing cavity formation. They managed to infiltrate the artificial caries lesion with adhesives in vitro with deeper penetration depth seen in the lesions pre-treated with acid conditioning [8].

A year later, Robinson et al. [9] used an experimental resorcinol-formaldehyde resin to occlude the pores of early enamel lesions. The resin occluded up to 90% of the pores after the second and third time of applications and simultaneously reduced the progression of the demineralisation. However, due to the potential cytotoxic hazard of the resin, mainly towards the vital dentine and pulp tissue, the use of the resin was discontinued [9,10]. The authors later suggested that the infiltrating resin should meet a certain standard: low viscosity, hydrophilic, anti-bacterial, non-toxic, aesthetically acceptable and able to polymerise into a solid state. The latter recommendation may give extra strength to the weakened enamel structure following demineralisation [10].

These two early experiments have opened the ‘floodgates’ to various in vitro and in vivo studies focusing on developing resins that can infiltrate and halt the progression of early enamel lesions. Earlier studies have used unfilled resin in the form of dental adhesives and sealants as the infiltrating agent. In vitro studies on an artificial caries model shows some potential, where all of the studies reported a reduction in caries progression as compared to the control [11,12,13]. In one study by Robinson et al. [11], they observed that 60% of the pores were occluded following the infiltration process. 

Clinical evaluations on the success of sealing the enamel lesion using sealants and adhesives among children were carried out by Gomez et al. [14] and later Martignon et al. [15]. While the latter reported a significantly reduced but still relatively high lesion progression (43.5%) over 18 months of observation, the former found no significant difference between the infiltrated and control lesion in their 24-month clinical study. It was suggested that the findings might be due to the disintegration of the material over time or inadequate sealing of the lesion [14]. This reflects the inability of the sealants and adhesives to penetrate deep into the lesion and, therefore, only showed superficial penetration into natural enamel lesions [16]. 

This led to a series of in vitro and in vivo studies led by Meyer-Lueckel and Paris [17,18] to develop a new material that does not disintegrate easily, has a good sealing ability and is able to penetrate deep into the base of the porous enamel lesion. The penetration ability of the material into a porous structure is best explained by the Washburn equation [19]. Based on the equation, the porous solid (in this context, the enamel structure) was regarded as a structure consisting of a collection of open capillaries, which can be penetrated by liquids driven by the capillary forces. Further, a material with high penetration coefficient (PC) can penetrate deeper and faster into the porous structure [20] and, upon hardening, can directly occlude and seals the porosity [17]. 

An earlier experiment conducted by Meyer-Lueckel and Paris [17] showed that materials with PC of more than 100 cm/s might be more suitable for application as ‘infiltrants’ for the treatment of early enamel lesions as compared to fissure sealants and adhesives. Furthermore, the same authors also found that resin materials with high triethylene glycol dimethacrylate (TEGDMA) concentrations tended to show better inhibition of lesion progression. This effect can probably be attributed to the better penetration capabilities of TEGDMA-based resins [17]. 

Their experimental resins using a mixture of TEDGMA, 2-hydroxyethyl methacrylate (HEMA), and ethanol showed the highest PC value, attributed to their low contact angle and low viscosity of these components. However, the mixture consisting of HEMA and ethanol showed inadequate hardening and was thus deemed unsuitable for infiltration of porous enamel. The mixture of resin monomer TEDGMA and ethanol, on the other hand, showed high PC value and sufficient hardening [18], suggesting that these two components would be the benchmark for the future development of RI. 

Further studies on the development of RI were based on the different formulations of TEDGMA and ethanol. Paris et al. [18] found that 99% of TEDGMA-containing resin gave a PC value of 204 cm/s, while the addition of TEDGMA and ethanol as solvents gave a PC value of 391 cm/s. The addition of solvent has been shown to increase the penetration coefficient and, consequently, the penetration depths, but inhomogeneities and uncured areas within the resin layer may occur, which can lead to incomplete polymerisation. Therefore, the authors suggest that the addition of ethanol to increase penetration abilities should be carefully balanced with the potentially impaired properties of the cured material.

They later concluded that low-viscosity RIs, based mainly on TEGDMA, had relatively high PC and were capable of inhibiting the progression of both artificial [21] and natural enamel caries lesions [22]. The high PC values also allow the material penetrates deep into the lesion body in a low-demineralising environment [23,24], and a 3 min application is sufficient to completely occlude the pores of the early enamel lesion [25]. 

### 2.2. Etching 

As described by the Washburn equation, the infiltration of resin into the porous enamel lesion is driven by capillary forces. The infiltration of the resin is strongly influenced by both the pore volume and the capillary radius of the solid to be penetrated. In the case of infiltrating early caries lesions, the presence of the highly mineralised surface layer with low pore volume might prevent the penetration of the infiltrating resin into the subsurface porosities underneath it [26]. Elimination of the surface layer to expose the porous subsurface lesion is desirable to aid the penetration of the resin. To achieve this, pre-treatment conditioning of the surface layer is needed prior to the infiltration process [16]. 

In restorative dentistry, acid etching has been identified as the core step in pre-treatment conditioning of the enamel surface prior to the placement of resin composite. Etching the enamel with 30–40% phosphoric acid resulted in selective demineralisation of the enamel prisms of varying irregularities and microporosities [27]. This allows the low-viscosity resins to ‘penetrate’ the microporosities, forming resin tags and micromechanical interlocking between the resins and the enamel [28]. Inspired by the success of the acid etching technique, a similar application might work for RI.

Grey and Shellis [29] successfully etched the artificial enamel caries with 36% phosphoric acid, which improved the penetration of the resin into the subsurface enamel lesion. Their in vitro results, however, cannot be replicated in the actual clinical scenario. Meyer-Lueckel et al. [30] found out that the penetration depth of the resin into the enamel porosities between the artificial and natural caries lesion was different. They postulated that the mineral content of the surface layer in natural caries lesions might be higher and more inhomogeneous due to the continuous cycles of de- and remineralisation in the oral cavity. They later concluded that complete erosion of the surface layer is necessary to expose the subsurface lesion prior to infiltration with low-viscosity resins. 

Two in vitro studies carried out by Meyer-Lueckel, Paris and their team [30,31] have demonstrated that in comparison to 37% phosphoric acid and 5% hydrochloric acid, the use of 15% hydrochloric acid gel for 90–120 s was effective in not only eroding the surface layer but led to nearly complete removal of the structure. This leads to exposure of the subsurface enamel lesion, which is ready to be infiltrated by the resin. However, without pre-treatment of the surface layer, penetration of resin was limited [29] and almost impossible for it to reach the subsurface enamel lesion. 

Elimination of the surface layer also can be performed mechanically, for instance, by using a diamond bur or polishing strip [32]. However, this procedure would cause occlusion of the lesion pores by smear layer, which later will affect the infiltration process of the resins. Furthermore, the reduction of the surface layer mechanically is difficult to control [30]. Thus, optimum surface erosion seems the best option in allowing the penetration of the low-viscosity resin into the subsurface enamel lesion effectively. 

### 2.3. Ethanol 

The use of ethanol as a solvent is believed to reduce the viscosity of the penetrating resin, as well as remove the remaining residual water that might be present at the bottom of the body lesion. Meyer-Lueckel et al. [16] found that the addition of ethanol enhanced the penetration ability of dental adhesives. Similar results were found with the experimental resin that they developed. The addition of 10% and 20% of ethanol had shown to increase the PC as well as the penetration depth [17,18]. 

Therefore, it can be assumed that a solvent like ethanol might affect the penetration behaviour positively. Alas, there is concern regarding the addition of the evaporating solvent that might possibly cause inhomogeneities and incomplete polymerised areas within the resin itself [18]. Paris et al. [33] conducted an in vitro study on extracted primary molars using experimental resins with and without the addition of ethanol. Although the PC value of ethanol-containing resin is higher, both the solvent-free and ethanol-containing resins managed to infiltrate the lesion completely. Hence, the authors suggest that concerning the potential effect of ethanol on the polymerisation of the RI, a solvent-free infiltrant is preferable [33]. In order to facilitate the removal of residual water and protein within the body lesion, the commercially available RI add-ons another step: pre-treatment of the enamel surface with ethanol following the acid etching procedure.

A summary of the main studies related to the development of the current RI is shown in Table 1. 

## 3. Present: Clinical Usage and Limitations of the Current RI

### 3.1. Arresting Non-Cavitated Early Enamel Lesion

The ultimate aim of developing RI is to delay the restorative procedure on a non-cavitated early enamel lesion. Placement of restoration will irreversibly remove the sound tooth structure surrounding the lesion and initiates the so-called ‘restorative cycles’ [34]. The use of fluoride varnish and casein phosphopeptide-amorphous calcium phosphate (CPP-ACP), coupled with good oral hygiene practise has been the mainstream in managing the non-cavitated early enamel lesion previously [35,36]. However, the remineralisation process requires time to be accomplished and is not immediate, highly dependent on the patient’s compliance and good oral hygiene measures [37] and unable to completely mask the white spot lesion [38,39].

RI, on the other hand, infiltrates the early enamel lesion using the low-viscosity resin monomer. The concept of this technique is based on the understanding of the structure and demineralisation process of the early enamel lesion itself. Following the demineralisation of enamel under an acidic challenge, minerals are dissolved out of the enamel, creating porosities within the enamel structure. These porosities act as a diffusion pathway for the dissolved minerals and acids. Infiltrating the early enamel lesion will occlude the pores and seals the lesion inside instead of at the surface. This will block the diffusion pathway of the organic acids, thus arresting the caries progression and inhibiting future demineralisation [17]. Further, infiltration also improves the mechanical strength of the porous enamel [24], resisting future demineralisation.

The clinical success of infiltrating early enamel lesions is evident in the literature, both for primary and permanent teeth. Infiltrating the non-cavitated early enamel lesion on the proximal surface has been the subject of many clinical trials. Ekstrand et al. [40], Page et al. [41] and Ammari et al. [42] reported a significant reduction in caries progression of the lesions on primary molars after infiltration with resin as compared to fluoride therapy. The therapeutic effects of RI over the fluoride varnish application were 21% higher over a two-year study period [41]. In addition, Urquhart et al. [43] reported that a combination of both RI and the application of 5% fluoride varnish on proximal early enamel lesions were five times more likely to cause caries arrest as compared to no treatment. Similar success was also reported in clinical trials on proximal lesions of permanent teeth, favouring the use of RI over fluoride therapy in managing non-cavitated proximal enamel lesions [44,45,46].

The performance of RI on the occlusal surface of teeth also has been investigated. Bakshandeh and Ekstrand [47] conducted a split-mouth study on the efficacy of RI in sealing the occlusal early enamel lesion of the primary molar tooth. Following an observational period of 22 months, the application of RI in combination with fluoride varnish significantly reduced the lesion progression as compared to the application of fluoride varnish alone. Later, Barakat and El-Soud [48] conducted another split-mouth study on children comparing the effectiveness of RI and compomer as a pit and fissure sealant material. They concluded that infiltration of the occlusal surface with resin provides a better retention rate as compared to compomer but harbours more bacteria due to the fluoride-releasing ability of the latter material.

The usage of RI in non-cavitated occlusal and proximal early enamel lesions is endorsed by the American Dental Association [49] in their evidence-based clinical practice guideline published in 2018. The guideline recommends the use of RI alone or in a combination of 5% fluoride varnish in arresting and reversing the non-cavitated lesion on the proximal surface, whereas for the lesion on the occlusal surface, a combination of RI and 5% fluoride varnish can be considered as an alternative to the conventional treatment of using fluoride varnish or fissure sealant. They believed that the relatively high cost of RI, as compared to sealants, might be the deterrent factor for its widespread use in community-based dentistry.

Further, two recently published systematic [50] and umbrella [51] reviews on the caries inhibition ability of RI on early enamel lesions showed that the relative risk for caries progression for the caries-infiltrated lesion is significantly reduced. Infiltrating the caries lesion is more effective for both primary and permanent teeth as compared to no treatment or other non-invasive preventive measures such as fluoride varnish, fissure sealant and oral hygiene instruction [50,51]. The authors also pointed out that the comparison of RI efficacy and caries lesion progression on primary and permanent teeth favours the successors, mainly attributed to the structural difference of enamel between the primary and permanent teeth. Lesion progression is quicker in primary teeth due to the fact that the enamel structure is less mineralised, more porous and thinner [50] as compared to permanent teeth.

Table 2 summerises the main studies on the ability of RI to arrest non-cavitated early enamel lesions.

### 3.2. Aesthetic Improvement of the Anterior White-Spot Lesion (WSL)

The ability of RI to infiltrate the non-cavitated early enamel lesion offers a new pathway and treatment modality in managing enamel WSL on the anterior teeth. The presence of the WSL is a result of enamel structure porosities, either due to enamel decalcification following the caries process or developmental disturbance during enamel formation. Clinically, the WSL appears as a chalky, whitish opacities surrounded by a normal translucent enamel due to the difference in optical density, measured as a refractive index between the normal and the porous enamel [52]. The subsurface demineralisation of the porous enamel retains more air (refractive index = 1.0) and water (refractive index = 1.33), causing higher light scattering as compared to the solid enamel structure (refractive index = 1.62). This led to a lower refractive index value that contributed to the abovementioned appearance. Poor aesthetic appearance [53], low self-esteem and negative psychosocial impact [54] have been reported in relation to the presence of WSL on anterior teeth among children.

Post-orthodontic decalcification is commonly associated with the presence of WSL on anterior teeth [55], where it increases the prevalence of WSL by three-fold as compared to those who were not wearing the appliance [56]. The presence of the orthodontic bracket and the archwire might hinder routine oral hygiene care and requires meticulous effort by the patient to maintain the area clean. Retention of dental plaque elevates the cariogenic bacterial count in the saliva, causing depletion in pH value at the dental plaque-enamel interface, thus initiating mineral loss from the enamel structure leading to enamel subsurface porosities [57,58]. The initiation of enamel decalcification can be seen as early as four weeks post orthodontic bracket placement [59].

Fluorosis and molar-incisor hypomineralisation (MIH) are the two most common developmental anomalies associated with WSL formation. Fluorosis occurs as a result of intoxication of the ameloblast cells caused by an excessive level of fluoride during the maturation stage of amelogenesis. In normal circumstances, the ameloblast alternates between smooth and ruffled-ended ameloblast, removing the matrix protein from the extracellular spaces and promoting enamel matrix mineralisation. However, excessive levels of fluoride disrupt the ameloblast cell function, causing amelogenin protein retention and areas of hypomineralisation within the enamel matrix [60,61]. This creates a subsurface enamel hypomineralisation and porosities, seen as WSL intra-orally.

MIH is a developmental defect characterised by hypomineralisation of systemic origin affecting one to all four first permanent molars and simultaneously might affect the permanent incisors as well [62,63]. A systemic and environmental insult towards the ameloblast during the maturation stage of amelogenesis is thought to be the culprit, leading to the formation of a hypomineralised area within the enamel structure. The exact aetiology of MIH is still unclear, but childhood illness, especially fever, asthma and pneumonia, maternal infection during pregnancy, consumption of amoxicillin during the first year of life, low birth weight and chicken pox infection are among the factors that have been proposed [64,65,66,67,68]. Clinically, the appearance of WSL on MIH incisors varies from creamy-white to yellow-brown opacities with well-demarcated borders [68] and may be accompanied by tooth sensitivity.

Reversal of the decalcification process (in the case of post-orthodontic decalcification) and improvement of the aesthetic value have been the aim of managing WSL on anterior teeth. Improvement in the child’s oral health and quality of life has been reported following the treatment of WSL [69]. Similar to the management of non-cavitated early enamel lesions, fluoride-based materials have been used as the first line of treatment in managing such cases [70]. In addition, microabrasion [71,72] and bleaching [73,74] also has been suggested to improve the aesthetic appearance of the WSL.

However, the masking ability and the outcome of these techniques are inconsistent as the aesthetic appearance remains impaired even after treatment [38,75], while the use of bleaching agents in the paediatric population is somewhat controversial, as the side effects of gingival irritation and sensitivity might not be well tolerated in young children. In Europe, the use of bleaching agents in children is restricted to 0.1% hydrogen peroxide [76], which is far from the effective concentration to produce a remarkable aesthetic improvement [77].

Infiltrating the WSL with resin allows the low-viscosity resin to penetrate the intercrystalline spaces of the hypomineralised and porous enamel. This resulted in an improvement in the refractive index value of the porous enamel (1.52), close to the value of normal enamel [78]. This has dramatically and instantly improved the appearance of the anterior teeth with WSL. High patient satisfaction has been reported following the infiltration of WSL [79,80]. Further, infiltration improves the surface microhardness [81] and shear bond strength while reducing the surface roughness of the infiltrated WSL [82].

In the literature, several systematic reviews concluded that there is inconclusive evidence to strongly support the use of RI in managing WSL on anterior teeth. This is, however, merely due to insufficient high-quality clinical trials rather than the clinical inefficiency of the technique itself. Most of the studies available were either laboratory-based, had a high risk of bias or were short-term studies, thus hindering the authors from making a conclusive recommendation [51,83,84,85]. All of the authors were in agreement regarding the need to conduct more high-quality clinical trials with long-term follow-ups in the future.

Nevertheless, based on the available clinical trials, positive camouflaging effects were reported on the WSL of anterior teeth treated with RI. Wang et al. [86] reported a favourable aesthetic outcome of RI over fluoride varnish among their orthodontic patients. Further, Knosel et al. [87], in their clinical study on post-orthodontic WSL, stated that the lesion showed significant aesthetic improvement and blended well with the normal enamel, even after a 24-months follow-up. Meta-analysis performed by Borouni and colleagues [85] showed a significantly higher optical improvement following infiltration as compared to the regular application of fluoride varnish. The author also stated that the masking effects of fluoride varnish might take up to six months, in contrast to the immediate masking effect of RI. In addition, two studies have been conducted by Gu et al. [71] and later Shan et al. [72] comparing the effects of RI and microabrasion in treating post-orthodontic WSL. They concluded that both techniques improve the aesthetic outcome of the WSL, but better and longer aesthetic outcomes were observed among the lesions treated with RI.

Treatment of WSL secondary to developmental defects using RI has also been the subject of clinical investigation. Clinical trials conducted by Gugnani et al. [74] reported an effective masking ability of RI on teeth with mild to moderate fluorosis, while Schoppmeier et al. [88] concluded that RI with increased etching and infiltration time gave significantly better aesthetic outcomes in comparison to bleaching. Gencer and Kirzioglu [89], in another study, concluded that treatment with RI on anterior teeth with fluorosis gave the highest colour changes as compared to fluoride varnish, CPP-ACP and microabrasion. This was in agreement with the clinical study conducted by Zotti et al. [79], as they observed the immediate disappearance of the fluorosis WSL following treatment with RI. Significant aesthetic satisfaction was reported by the patients where the changes remained stable during their one-year follow-up.

A recently conducted systematic review also favours RI over microabrasion and bleaching in the management of fluorosis [90]. The author further suggested a combination of in-house bleaching and RI for a better aesthetic outcome. However, Borouni et al. [85] disagreed with the recommendation. Through their meta-analysis, pre-treatment of the fluorosis teeth with bleaching before infiltration did not give any significant masking effects as compared to infiltration alone. Further, the safety usage of bleaching agents among paediatric patients should be evaluated beforehand to avoid unnecessary iatrogenic damage.

RI was also used in the management of anterior teeth with MIH [91,92]. Earlier studies by Kim et al. [93] found that the MIH teeth treated with RI showed significant colour changes after one week of treatment. However, 40% of the infiltrated teeth remained unchanged. They postulated that the failure was associated with the depth of the lesion, which is beyond the penetration ability of the resin, and the highly mineralised surface layer, which had not been eroded long enough to achieve the desired effect. Repeated application of acids to encourage more surface erosion might be able to improve the infiltration efficacy in such lesions. This is further supported by Somani et al. [94]. They stated that variation in the depth of the lesion plays an important role in deciding which treatment protocol should be chosen for a different type of MIH lesion. RI showed some potential and can be effective in reversing the white opacities, provided that the surface layer has been removed sufficiently.

Elbaz and Mahfous [95] and Bhandari et al. [96] both investigated the effect of RI in mild MIH-affected incisors. Both studies reported the efficacy of the RI technique in masking the opacities and improving the aesthetic appearance of the incisors. The former author also observed the superior ability of the RI technique over 5% sodium fluoride is masking the WSL on incisors with mild MIH. In addition, Hasmun et al. [69] used a combination of microabrasion and RI in managing MIH-affected incisors in 23 children. They used digital clinical images as the tools, comparing the pre-and post-treatment effects after six months. The author observed a significant reduction in brightness and surface areas affected by the opacities. They later concluded that the technique is efficient in reducing the incisor opacities related to MIH.

The European Association of Paediatric Dentistry (EAPD) [68], in their recently updated policy document on best clinical practice in managing MIH, suggested that RI can be considered as one of the treatment options in managing MIH-affected incisors, either alone or in combination with other treatments including microabrasion, external bleaching and direct composite restoration, depending on the severity and depth of the lesion.

The main studies on the ability of RI to improve the anterior WSL are shown in Table 3.

### 3.3. Limited Penetration Depth

One of the drawbacks of RI is the limited penetration depth of the material into the carious lesion. Despite various systematic reviews describing the efficacy of RI infiltrating the early enamel lesion, the penetration of the low-viscosity resin is limited to the enamel structure and did not perform well in the dentinal lesion. Peters et al. [45] reported that while the resin managed to infiltrate 100% into the inner enamel lesion (E2), only 64% of the outer dentine lesion (D1) was infiltrated by RI.

The histological difference between the enamel and dentinal carious lesion was thought to be the reason behind the poor penetration ability of the resin. The dentinal tubules, upon demineralisation, will be enlarged, creating a pathway for the outflow of the dentinal fluids into the lesion. This later prevents a complete penetration of the resin into the dentinal lesion [97].

Schneider et al. [98] studied the penetration ability of RI on extracted teeth using microcomputed tomography. They concluded that the resin was incapable of completely infiltrating the advanced carious lesion, with areas of resin inhomogeneity found within the lesion. Several in vitro studies on bovine and extracted premolar teeth reported the mean penetration depth of the RI to be less than 70% [99,100,101]. In different ICDAS codes, better penetration was reported in enamel lesions (ICDAS code 2–3) in contrast to the deeper lesion [102]. This was further supported by a systematic review and meta-analysis conducted by Liang et al. [103] and later Soveral et al. [82], where both authors described the limited ability of the resin to infiltrate deep into the dentinal lesion.

The penetration depth of RI in primary teeth is better as compared to the permanent teeth. Soviero et al. [104] reported a penetration depth between 70 to 80% in deciduous molars viewed under the scanning electron microscope, while although not significant, Aswani et al. [105] also observed higher penetration ability of the material into the carious lesion of primary teeth. The difference is attributed to the difference in the enamel structure between the two types of dentition, where deciduous enamel is less mineralised and thinner, providing a better platform for the resin to infiltrate the lesion faster and deeper.

The limited penetration depth of the resin will have some clinical implications. Liang et al. [103] suggested that the clinician should appropriately select which of the non-cavitated carious lesion is suitable for RI, guided by the depth of the lesion. This is also true for deeper WSL found on incisors teeth diagnosed with MIH. Due to the variety in the lesion depth of MIH-affected incisors, the masking ability of the RI might differ between cases; thus, case selection is very important to ensure an optimum aesthetic result.

### 3.4. Not Radiopaque

Ideally, a dental material should possess a certain degree of opacity in the radiograph. It is of paramount importance for the clinician to be able to distinguish the material against the surrounding background of enamel and dentine. This allows the clinician to assess the quality and clinical performance of the material. Apart from that, the presence of secondary caries, microleakage, marginal adaptation and overhangs can be detected clearly in the radiograph [106]. International Standard Organisation (ISO) recommends a dental material should have a radiopacity equivalent to or greater than the radiopacity produced by the thickness of pure aluminium [107].

However, this is not the case with RI. The RI is radiolucent in the radiograph, showing no clear distinction between the material and the infiltrated enamel and dentinal lesion. This will cause problems for the clinician as the penetration depth of the resin would not be visible radiographically. Further, the potential caries progression of the infiltrated lesion could not be monitored. Instead, multiple radiographs need to be taken periodically, and comparisons between radiographs need to be made for the sake of monitoring the success or failure of the infiltration technique. In addition, medico-legal issues, such as a dispute over the treatment charges, may arise. However, the clinician would face difficulty in proving that the carious lesion has been infiltrated with RI due to the radiolucency of the material [108].

This ‘loophole’ is worth to be explored. Thus, it is not surprising that the development of RI with radiopaque properties has been conducted actively by some researchers, which will be discussed in the next section.

### 3.5. Long-Term Colour and Material Stability

Aesthetic demand by parents and teenagers has increased over the years. The demands are even escalated when there are discrepancies in terms of colour, shape and alignment involving their teeth. With regards to restoring the anterior teeth, the colour stability of the resin-based material is one of the components that determine the long-term success of the material [109,110,111].

RI has been associated with poor long-term colour stability [109]. The infiltrated teeth are unable to retain the masking effects and are susceptible to staining by the colouring agents present in the diet, as proved by various in vitro studies. Significant colour changes have been reported following immersion with coffee [110,111], tea [110], red wine [112,113] and grape juice [114]. The colour changes were also more apparent in resin-infiltrated teeth as compared to teeth treated with remineralising agents [115]. The staining effects may be influenced by the duration of exposure and the intensity of the colouring [109].

The composition and ‘behaviour’ of the resin have been identified as one of the factors that contribute to the problem. TEDGMA has been proven to have the highest penetration coefficient as compared to others. However, TEDGMA also shows a high degree of water absorption owing to the presence of ether bonds which are said to be hydrophilic in nature [116]. In addition, the resins present with low filler content, which subjects the material to a certain degree of polymerisation shrinkage. The coefficient of thermal expansion also differs between the enamel structure and the resin. Over time, this unsynchronised expansion can lead to the formation of microcracks. Microcracks and material shrinkage increased the tendency of the material to absorb water as the material continued to degrade over time. This leads to discolouration of the material and subsequently affects the aesthetic outcome of the treated teeth [110].

Surface roughness also has been implicated as the cause of staining, mainly involving resin-based restoration [112]. The rough surface can act as the niche for dental plaque and bacterial adhesion, including stains and colouring from the diet. The minimal limit for acceptable surface roughness is valued at around 200 nm. Beyond this, plaque accumulation is inevitable [117]. Under vicious cycles of pH alteration, the bacterial and plaque accumulation subsequently cause degradation of the resin material, demineralisation of the enamel and potential formation of secondary caries [118]. Changes in surface integrity increase the tendency of the infiltrated teeth towards water absorption, which leads to surface staining and loss of surface gloss [119]. This has been proven by a number of in vitro studies conducted using bovine and extracted human teeth. Most of the authors were in agreement as they observed increased surface roughness and higher discolouration of the infiltrated teeth, even after polishing, as compared to sound enamel structure [110,118,119].

Despite the long-term colour instability, repolishing the surface of infiltrated teeth has improved the brightness of the discoloured surface [112,120]. Paris et al. [78] suggested that clinicians should wipe off the excess resin from the surface before light-curing to prevent excess material from sticking on the enamel surface, potentially attracting plaque accumulation. Further, the same authors also advocate polishing the surface post-infiltration. In addition, bleaching of the infiltrated surface has been suggested to improve discolouration [116,121]. The latter author also stated that surface bleaching did not interfere with the caries-arrest ability of RI. However, the use of bleaching agents among the paediatric population should be treated with caution to prevent unwanted iatrogenic damage to the gingiva and tooth structure, as described earlier.

Interestingly, clinical studies of resin-infiltrated anterior teeth did not report any significant discolouration or staining effects post-infiltration. Nevertheless, most of the clinical studies were of short-term follow-up, with the longest follow-up period reported by Knosel et al. [87] at 24 months. Meyer-Lueckel et al. [122] and Paris et al. [46] reported a longer follow-up period of four and seven years, respectively, for the infiltrated lesion on the proximal surface. However, colour changes were not part of their assessment, most probably due to the lesion located in the non-aesthetic zone. Further, the patient is brushing efficiency and frequency of dietary intake, in this case, those that can cause surface discolouration, such as coffee as well as a smoking habit, may play a role with regards to the staining tendency of the infiltrated surface. Thus, a true reflection on the colour stability of the resin-infiltrated anterior teeth could not be made. Despite that, the clinician should warn the parents and patient regarding the potential staining effects of the resin-infiltrated teeth and advice them for regular polishing and avoidance of certain foods and beverages that could stain the tooth structure if possible. Educating the parents and patients is the key element in paediatric dentistry, which helps the clinician in managing their expectations and avoidance of future disputes over the treatment provided.

## 4. Future: Innovation to Improve the Current RI

### 4.1. RI with Remineralising and Antibacterial Properties

Incorporation of an antibacterial or remineralising agent into the current RI formulation will offer huge advantages to the patients. While the former prevents bacterial colonisation at the infiltrated enamel surface, the latter enhances the remineralisation of the lesion and improves the hardness and resistance of the lesion toward a future acidic challenge [123]. With regards to Paediatric Dentistry, this is highly desirable and advantageous, especially in high-caries risk children where the progression of the lesion can be halted immediately, and more focus can be shifted to improving the child’s oral care habits and other preventive measures.

Bioactive glass (BAG), hydroxyapatite particles (HAP) and calcium phosphate (CaP) in the form of fillers are among the remineralising agents that have been incorporated in various dental restorative materials with great success. Apart from its anticaries activities, it also reduces the polymerisation shrinkage of the resin-based material. Similar concepts and ideas can be adopted in the formation of a RI with remineralising and antibacterial properties. One problem that might arise following the incorporation of fillers within the RI monomer matrix is the increase in viscosity and stiffness of the material, which will affect the penetration ability of the resin. It was suggested that the concentration of filler within a resin-based dental material should not exceed 15% of the total weight ratio [124]. However, with the advancement in the field of nanotechnology, a nano-sized filler can be formulated. This will allows the incorporation of the remineralising and antibacterial agent into the RI monomer matrix without affecting its penetrative ability.

Hashemian et al. [125] investigated the penetration ability, mechanical properties and calcium release ability of their experimental RI consisting of 10% polyurethane acrylate oligomer (PUA) and 88% TEDGMA. The resin showed a good penetration coefficient of 133 cm/s, exceeding the minimum threshold of 100 cm/s required for effective penetration of resin into the enamel porosity. The penetration depth was up to 120 µm. The authors later added either HAP or BAG as filler into the formulation of their experimental resin. The incorporation of both fillers increased the microhardness and modulus of elasticity of the RI, as well as water sorption and solubility of the material. The latter outcome might reduce the colour stability of the material, although the degree of conversion (DC) did not significantly differ from the commercial RI. The DC value here is closely related to the water sorption and solubility of the material, where low DC is correlated with high water sorption and solubility, which will contribute to the poor clinical performance of the material [126]. Further, the author reported that the addition of HAP is more homogenous but releases fewer calcium ions which contribute to lower demineralisation resistance as compared to BAG-added RI.

A similar outcome was observed by Prodan et al. [127] using the fluoride-releasing glass filler, where incorporation of the filler improves the DC and strength of the material and inhibits demineralisation through the fluoride-releasing effect. However, contrasting findings were reported by Sfalcin et al. [128] in terms of water sorption and solubility of their experimental resin containing 25% ethoxylated bisphenol A dimethacrylate (BisEMA) and 75% TEDGMA doped with either HAP and BAG. In their study, the addition of either HAP and BAG produced lower water sorption and solubility and significantly higher DC value, microhardness and modulus of elasticity as compared to the commercial RI. This promising outcome proved that the use of HAP and BAG as fillers in the RI system carries the potential to improve the mechanical properties and caries arrest ability of the existing RI.

The use of CaP as fillers in RI was the subject of investigations conducted by Sfalcin et al. [128], Dai et al. [129] and Obeid et al. [130]. Sfalcin [128] and Dai [129] incorporated the amorphous CaP particles into their respective experimental RI monomer by the weight ratio of 10% and between 10 to 30%, respectively, while Obeid et al. [130] used nanofibers of polylactic acid (PLA) filled with CaP incorporated into silica network (PLA-SiO_2_-CaP) as their filler material. The addition of CaP increased the surface hardness and the DC value of the RI, and positive calcium and phosphate ion release were observed under pH-cycling challenge for PLA-SiO_2_-CaP and RI-30% CaP, respectively [129,130]. The deposition of interprismatic mineral crystal was observed by Dai et al. [129], suggesting the remineralising potential of the material. Interestingly, the addition of CaP increased the water sorption and solubility of the RI as compared to HAP- and BAG-added RI, respectively [128].

Apart from that, the effects of the incorporation of antibacterial agents as fillers on the mechanical properties and caries arrest ability of the RI also have been reported in the literature [97,131,132,133,134,135,136]. Kielbassa et al. [97] investigated the effectiveness and penetration ability of silver-enhanced RI. Silver, which is well-known for its antibacterial effects, was incorporated into the RI in the form of nanosilver particles (AgNP). Using the innovative tunnelling approach, the use of the experimental resin as an external infiltrant penetrated the proximal dentinal lesion and occluded the pores, similar to the commercial RI, suggesting that the addition of silver did not interfere with the penetration ability of the material. Further, the presence of silver may enhance the antibacterial effects of the material.

Another innovative antibacterial infiltrant was described by Cuppini et al. [135]. They used the ionic liquid 1-n-butyl-3-methylimidazolium bis(trifluoromethanesulfonyl)imide (BMI.NTf_2_) encapsulated with methacrylate-based microcapsules as the RI fillers. BMI.NTf_2_ was previously used as an antibacterial coating on polymers and implant surfaces [137], showing good antibacterial effects against *S. mutans* [138]. The addition of the fillers did not alter the handling and mechanical behaviour of the material, represented by the similar tensile strength of the commercial and experimental RIs. Further, the addition of 5% and 10% by weight ratio of the fillers reduced the surface free energy (SFE) and increased the contact angle of the material. This subsequently increases the hydrophobicity and reduces the wettability of the resin, improving its hydrolytic stability in the oral environment. This feature also has antibiofilm effects, as increased contact angle and low SFE could impair bacterial adherence and colonisation [139]. However, the penetration ability and other mechanical properties of the ionic liquid-loaded RI were not assessed by the authors.

The use of chlorhexidine diacetate salts [132], iodonium salts [133], quaternary ammonium-based resin monomer [134] and guanidine hydrochloride [140] were also being investigated as potential antibacterial RI fillers. The addition of chlorhexidine and iodonium salts improved the DC and microhardness of the experimental resin and inhibited the *S. mutans* and *L. acidophilus* biofilm, while the incorporation of the quaternary ammonium-based resin monomer at 2.5% to 10% by weight ratio reduced the *S. mutans* bacterial count without affecting the surface roughness of the material. In addition, the incorporation of guanidine hydrochloride as filler of RI was also effective in disrupting the growth of *S. mutans* biofilm without affecting the SFE, DC and contact angle of the material. On the other hand, Skuca-Nowak et al. [131] and Fisher et al. [136] used metronidazole, an antibiotic that is highly effective against anaerobic bacteria and protozoa mixed with a methacrylate monomer (PMMAn) as the RI filler. The experimental resin managed to penetrate the demineralised enamel lesion up to 40 µm as well as the demineralised root cementum and dentine. However, the relevance of using metronidazole might be questioned as the antibiotic is less effective against cariogenic bacteria.

Conclusively, the addition of remineralising and antibacterial agents as the nano-sized filler has opened a new prospective in the development of a RI with enhanced anticaries properties. Although most of the studies were in vitro based, and some did not evaluate the penetration ability of the material on enamel lesion, the potential of those materials are massive and promising. The path has been laid, and with further research and clinical trials, it is just a matter of time before the products based on this concept hit the market.

### 4.2. Self-Etching RI

The whole process of infiltrating a non-cavitated carious lesion involves at least five major steps, which include the isolation, application of acid etching, ethanol and dual-application of RI with a 40-s light curing procedure in between them. The commercial RI uses 15% hydrochloric acid as surface pre-treatment before infiltrating the carious lesion with resin. This will sufficiently erode the surface layer and allows the penetration of the resinous material into the body lesion. The idea of developing a self-etching RI is well appreciated, especially in the field of Paediatric Dentistry. This would cut down the lengthy procedure, providing better management of the paediatric patient, especially those with short-term attention spans [141].

Wang et al. [142] have investigated the penetrative ability of their experimental self-etching RI, which consists of the resin monomer TEDGMA and phosphoric acid 2-hydroxyethyl methacrylate ester (PAM). Using the artificial WSL on bovine teeth, the composition of 75% TEDGMA: 25% PAM showed some potential as the penetration depth was found to be similar to the control when no acid etching was performed prior to the infiltration procedure. The experimental resin also demonstrated acceptable viscosity, which allows the material to penetrate the lesion.

The author stated that there is an inverse relationship between the ratio of PAM and the pH of the material, whereas, as the ratio of PAM increases, the pH becomes more acidic, but the material becomes more stiff and viscous. This will reduce the penetration ability of the resin and prevents it from infiltrating deeper lesion. Thus, they suggested the ratio of 75% TEDGMA: 25% PAM as the best ratio to be used as a self-etching RI.

Nevertheless, this product is still in its preliminary stages, and further research has been recommended by the author to explore its potential in infiltrating the WSL under different experimental settings.

### 4.3. Radiopaque RI

As mentioned in the previous section, the radiolucent appearance of the infiltrated lesion in the radiograph causes difficulty for the clinician to monitor the success or progress of the lesion. Therefore, the development of a RI with radiopaque properties is highly desirable. Moeinian [108] investigated the use of strontium hydroxyapatite (SrHA) nanoparticles and strontium bioglass (SrBG) as a filler mixed into the resin monomer TEDGMA. The author found out that the mixture resulted in a below-average radiopacity and increased the viscosity of the RI, thus limiting its potential to infiltrate the carious lesion.

The author later used barium and tin methacrylate monomers as potential radiopaque material. The incorporation of both radiopaque resin monomers produced a homogenous mixture with TEDGMA with low viscosity and contact angle values. The experimental RIs were later used to infiltrate artificially-created WSL. Although both mixtures managed to infiltrate the lesion to a certain depth, they were not clearly visible in the digital radiograph. The author concluded that despite meeting the required standard criteria of radiopacity, both experimental resins produced radiopacity lower than enamel, making it undiscernible in the radiograph. Increasing the volume of the radiopaque resin monomer is not possible as it would impair the mechanical properties of the RI [108].

Efforts in formulating a radiopaque RI were also made by Pedreira et al. [143] and Nowak-Wachol et al. [144]. The former authors investigated the radiopacity potential of an experimental RI, using either barium or zirconium oxide particles which served as a filler and incorporated into the RI monomer. In addition, the water sorption, solubility and penetration depth of the experimental resin were also evaluated. Their in vitro study showed that the mixture of 45% zirconium oxide particles (by weight) with RI monomer produced radiopacity higher than enamel when viewed using a digital radiograph. Further, this experimental resin demonstrated a comparable penetration depth but lower water sorption and solubility when compared to the commercial RI, suggesting its promising potential in dentistry. On the other hand, the mixture of 25% barium oxide particles (by weight) with RI monomer did not perform well for all the investigated outcomes [143].

Nowak-Wachol et al. [144], on the other hand, used yttrium trifluoride (YF_3_) incorporated in their experimental RI consisting of resin monomer TEDGMA, HEMA and a bioactive methacrylate monomer, PMMAn with build-in metronidazole. They hypothesised the potential antibacterial and radiopaque effects of the experimental RI following the addition of YF_3_. However, the penetration of the experimental resin into the carious root dentine was hampered by the inability of YF_3_ to penetrate deeper into the lesion due to its large particle size.

The incorporation of 45% (by weight) zirconium oxide showed some potential and is by far the best option available at the moment. It truly has shed some light on the efforts of producing a radiopaque RI. Nevertheless, since it was conducted on an in vitro basis, whether the zirconium-enhanced RI would perform well in the in vivo or ex vivo setting is still under investigation. Further research is still needed to prove the potential of this material.

### 4.4. Micro-Filled RI

The current RI is highly effective in arresting the non-cavitated enamel lesion by occluding the pores and blocking the diffusion pathways of the minerals and acids. However, as pointed out before, the penetration ability of the RI is limited to the enamel lesion only with the ICDAS score of 2 and 3 [96], and the performance in arresting deep dentinal carious lesions is poor, with less than 65% of the outer dentinal lesion (D1) were completely infiltrated [45]. This failure is attributed to no filler being incorporated within the RI matrix monomer, contributing to high polymerisation shrinkage and lack of ‘bulk’ of the material.

This has led Meyer-Lueckel and Paris [145,146], together with their innovative research team members, to conduct a series of experimental studies investigating the possibility of incorporating fillers into the RI monomer matrix without jeopardising its penetrative ability. In their experimental study, two organic fillers of different particle sizes and one glass filler at 0.7 µm were mixed with the RI monomer TEDGMA, reaching up to 55% of filler content. The percentage of lesion penetration for each formulation was assessed using the artificial enamel caries model with confocal laser scanning microscopy. The outcome showed that the filler size influenced the penetration ability of the resins, where RI with 42 µm of organic filler displayed an indifferent penetration percentage as the commercial unfilled RI [145]. The success of this early experiment has paved the way for the improvisation of the current RI formulation and indirectly improved the mechanical properties of the material as well.

Subsequently, using a similar formulation, the micro-filled RI was applied to the natural enamel carious lesion of extracted teeth with ICDAS scores of 2, 3 and 5. The latter score indicates a cavitated carious lesion that has extended into the dentine, with minimal loss of enamel structure. The micro-filled RI showed similar efficacy in penetrating the natural enamel carious lesion as the commercial unfilled resin but managed to fill the ICDAS 5 cavity as assessed through the dual-fluorescence staining and confocal microscopy [146].

The presence of the organic filler has given more ‘bulk’ to the RI, contributing to the success of the newly formulated material in filling up the small cavity with less polymerisation shrinkage. However, the authors did not assess the compressive strength of the micro-filled RI and compared it with the conventional resin composite or glass ionomer restoration. It is also interesting to see whether this micro-filled RI can be used as a fissure sealant and restoration of small abrasion cavities. If proven, this certainly will add more versatility to the material, supporting the micro-invasive technique in the management of carious enamel lesions in children. Further, the performance of the material in a clinical setting is yet to be determined and warrants more clinical trials in the future. Despite that, the future of RI in Paediatric Dentistry is definitely bright and promising.

A summary of the main laboratory-based studies conducted to improve the current RI is shown in Table 4.

## 5. Conclusions

RI has emerged as one of the important micro-invasive techniques in addressing non-cavitated and white-spot enamel lesions in children and adolescents. However, some issues of concern have been raised over the radiopacity, colour stability and its usage in deep dentinal lesions. Improvisation of the current RI is ongoing actively at the moment, aiming to address the clinical issues associated with the material. Nevertheless, most of the studies are laboratory-based, which might lead to different outcomes clinically. Thus, further research in terms of clinical trials is needed.

### Limitations

This narrative review was conducted by screening through all the relevant studies in the literature to support the ideas and arguments that we have pointed out in this review. This includes umbrella and systematic reviews, randomised clinical trials, non-randomised clinical studies and laboratory-based studies. The level of bias and strength of the studies were not assessed. As discussed before, although the value of laboratory-based studies cannot be denied, their impacts and effects in the actual clinical setting might be different.

## Figures and Tables

**Table 1:** Summary of main studies related to the development of RI.

Resin Monomer and Ethanol		
Study	Materials	Summary of Findings
Paris et al., 2007 [17]	Dental adhesives: Excite, Vivadent, Schaan, Liechtenstein.	Excite was not able to penetrate the lesion body completely. Resin with TEDGMA and ethanol gave the highest PC, while BisGMA affected the PC negatively. Materials with PC > 100 cm/s showed better penetration and were suitable for development as infiltrants.
	Experimental resin monomer: BisGMA, TEDGMA and ethanol (Sigma–Aldrich, Steinheim, Germany) of different percentage	
Paris et al., 2007 [18]	Experimental resin monomer: BisGMA, TEDGMA, HEMA and ethanol (Sigma–Aldrich, Steinheim, Germany) of different percentage	The addition of ethanol significantly increased the PCs due to a decrease in viscosity and contact angle to the enamel. The highest PCs were found for mixtures containing TEGDMA, HEMA and 20% ethanol.
Meyer-Lueckel and Paris 2008 [21]	Experimental resin monomer: BisGMA, TEDGMA, HEMA and ethanol (Sigma–Aldrich, Steinheim, Germany) of different percentage	Progression of artificial carious lesion depth was negatively correlated to PC and application time Materials with high PC value inhibited the lesion progression
Meyer-Lueckel and Paris 2008 [22]	Experimental resin monomer: BisGMA, TEDGMA, HEMA and ethanol (Sigma–Aldrich, Steinheim, Germany) of different percentage	Experimental resins with higher PCs showed better penetration ability as compared to resins with lower PCs in the natural caries lesion model.
Meyer-Lueckel and Paris 2010 [23]	Experimental resin monomer: BisGMA, TEDGMA and ethanol (Sigma–Aldrich, Steinheim, Germany) of different percentage	Material with high PC is able to penetrate the lesion body completely. Solvent-free resin, mainly consisting of TEDGMA, seems to be preferable.
Paris and Meyer-Lueckel 2010 [24]	Experimental resin monomer: TEDGMA and ethanol (Sigma–Aldrich, Steinheim, Germany)	Resin infiltration is effective in preventing further demineralisation of artificial enamel caries lesions under cariogenic conditions in situ
Meyer-Lueckel et al., 2011 [25]	Experimental resin monomer: TEDGMA and ethanol (Sigma–Aldrich, Steinheim, Germany)	3 min application of an infiltrant was sufficient to achieve almost complete penetration of enamel caries.
Paris et al., 2012 [33]	Experimental resin monomer: BisGMA, TEDGMA and ethanol (Sigma–Aldrich, Steinheim, Germany) of different percentage	Solvent-free and ethanol-containing resins infiltrated the natural enamel lesion completely. The addition of ethanol or resins with higher PC values did not result in significantly deeper penetration.
**Etching**		
Meyer-Lueckel et al., 2007 [30]	37% phosphoric acid gel 5% and 15% hydrochloric acid gel	Etching with 15% hydrochloric acid gel for 90–120s effectively eroded the surface layer of natural enamel caries.
Paris et al., 2007 [31]	37% phosphoric acid gel 15% hydrochloric acid gel	Etching with 15% hydrochloric acid gel is more suitable than 37% phosphoric acid gel as a pre-treatment for caries lesions intended to be infiltrated.

TEDGMA—triethylene glycol dimethacrylate; BisGMA—bisphenol A-glycidyl methacrylate; HEMA—2-hydroxyethyl methacrylate; PC—penetration coefficient.

**Table 2:** Summary of main studies on the ability of RI to arrest non-cavitated early enamel lesions.

Randomised Controlled Trials			
Study	Comparisons	Follow-Up Period	Outcomes
Ekstrand et al., 2010 [40]	FV (control); the proximal surface of primary teeth	One year	RI, in combination with FV, gave a better outcome in controlling proximal lesion progression on primary molar teeth as compared to fluoride varnish alone.
Page et al., 2017 [41]	FV (control); the proximal surface of primary teeth	Two years	RI is more effective than FV in controlling the progression of the proximal carious lesion in primary molars Most children find the treatment acceptable
Ammari et al., 2018 [42]	Flouride toothpaste and flossing (control); the proximal surface of primary teeth	One year	RI of proximal caries lesions in primary molars is significantly more efficacious in inhibiting lesion progression than standard therapy alone (fluoride toothpaste + flossing).
Meyer-Lueckel et al., 2012 [44]	Fluoride therapy and flossing (control), the proximal surface of permanent teeth	Three years	RI of proximal caries lesions in permanent molars can be said to be more efficacious in reducing lesion progression as compared to standard fluoride therapy and flossing.
Peters et al., 2019 [45]	Periodic prophylaxis and fluoride therapy; the proximal surface of permanent teeth	Three years	RI recorded high efficacy with 71% of relative risk reduction as compared to control. Radiographically, RI was 100% successful in arresting caries progression in inner enamel lesions (E2) and 64% in outer dentin lesions (D1).
Paris et al., 2020 [46]	Fluoride therapy and flossing (control), the proximal surface of permanent teeth	Seven years	The relative risk reduction for the test in relation to control was 80%. RI is efficacious in reducing lesion progression of proximal caries lesions on permanent molars extending around the enamel dentin junction.
Bakshandeh and Ekstrand 2015 [47]	FV and FS, the occlusal surface of primary teeth	Three years	Infiltrating or sealing occlusal surfaces in combination with FV application on initial occlusal caries lesions of primary molar teeth showed high efficacy in arresting caries progression as compared to FV alone.
Barakat and El Saud 2022 [48]	Compomer, the occlusal surface of primary teeth	Six months	RI retained longer on the occlusal surface of primary teeth as compared to the compomer. Compomer has a lower bacterial load due to its fluoride-releasing properties.
**Systematic/umbrella reviews**			
**Study**	**Outcomes**		
Urquhart et al., 2019 [43]	Systematic review and meta-analysis A combination of both RI and application of 5% FV on proximal early enamel lesions were five times more likely to cause caries arrest as compared to no treatment		
Faghihian et al., 2019 [50]	Systematic review and meta-analysis RI significantly reduced the risk and prevented initial caries progression in primary and permanent teeth.		
Lin et al., 2022 [51]	Umbrella review RI showed favourable outcomes in minimizing the risk of caries progression of WSL, more in permanent as compared to primary teeth. Due to limited data, an analysis of the aesthetic outcome was unable to perform.		

RI—resin infiltration; FV—fluoride varnish; FS—fissure sealant; WSL—white spot lesion.

**Table 3:** Summary of main studies on the ability of RI to improve the anterior WSL.

Randomised Controlled Trials				
Study	Type of WSL	Comparisons	Follow-Up Period	Outcomes
Wang et al., 2013 [86]	Post-orthodontic WSL	FV	6 months	Significant improvements in the WSL were observed following treatment with both RI and FV. RI gave better aesthetic effects as compared to FV.
Knosel et al., 2019 [87]	Post-orthodontic WSL	No treatment	24 months	Assimilation of infiltrated WSL to the colour of adjacent enamel was observed. The camouflaging effects achieved by RI were stable, with no significant changes over at least 24 months.
Gu et al., 2019 [71]	Post-orthodontic WSL	Microabrasion	12 months	RI and microabrasion improved the esthetic appearance of WSL. RI showed a better aesthetic improvement effect when compared with microabrasion at 12 months.
Shan et al., 2021 [72]	Post-orthodontic WSL	Microabrasion	6 months	RI and microabrasion were both effective in reducing the sizes of WSL. RI showed better aesthetic outcomes over microabrasion.
Gugnani et al., 2017 [74]	Fluorosis	Bleaching with 37% H_2_O_2_, RI alone or RI + bleaching	1 year	RI groups (alone or in combination with bleaching) gave significantly better aesthetic results as compared to bleaching alone. Treatment of RI with increased etching and infiltration time gave the best outcome.
Schoppmeier et al., 2018 [88]	Fluorosis	RI alone or in combination with 25% H_2_O_2_	6 months	RI alone effectively masked mild to moderate WSL of dental fluorosis. Bleaching with 25% H_2_O_2_ before RI treatment provides significantly better masking effects.
Gencer and Kirzioglu 2019 [89]	Fluorosis	FV, microabrasion, CPP-ACP	6 months	RI and microabrasion, followed by FV and CPP-ACP, improve the aesthetic outcome of the treated teeth. RI gave the highest lightness value and was more effective in the treatment of fluorosis.
Zotti et al., 2020 [79]	Fluorosis	No treatment	1 year	The disappearance of WSL was observed after initial treatment. Significant aesthetic satisfaction was reported by the patients Changes remained stable at 1-year follow-up.
Kim et al., 2013 [93]	MIH	No treatment	1 week	RI managed to mask the discolouration of MIH-related WSL, while 40% of the lesion remained unchanged.
Elbaz and Mahfouz 2017 [95]	MIH	FV	1 month	RI was significantly better than FV in masking the WSL in children with (MIH).
Bhandari et al., 2018 [96]	MIH	No treatment	6 months	RI showed immediate aesthetic improvement in mild MIH-affected incisors The reduction of WSL remains stable after 6 months.
Hasmun et al., 2020 [69]	MIH	Microabrasion, bleaching, composite restoration	6 months	The combination of microabrasion and RI caused a significant reduction in brightness and surface area affected by MIH.
**Systematic/umbrella reviews**				
**Study**	**Type of WSL**	**Outcomes**		
Borges et al., 2017 [83]	Post-orthodontic WSL	Systematic review Only two RCT studies were regarded as low risk of bias. There is no strong evidence to support this technique based on the present clinical studies.		
Sonesson et al., 2017 [84]	Post-orthodontic WSL	Systematic review The quality of evidence was graded as very low. There is a lack of reliable scientific evidence to support remineralizing or camouflaging strategies to manage post-orthodontic WSL.		
Borouni et al., 2021 [85]	Post-orthodontic WSL and fluorosis	Systematic review and meta-analysis There was a moderate level of evidence showing RI had a significantly higher optical improvement on post-orthodontic WSL as compared to no treatment or treatment with FV. A moderate level of evidence showed that bleaching prior to resin infiltration of fluorosis gave no difference in the masking effect as compared to infiltration alone.		
Lin et al., 2022 [51]	Post-orthodontic WSL	Umbrella review Only three studies regarded as high-quality Qualitative synthesis suggested that resin infiltration demonstrated acceptable aesthetic results Quantitative umbrella analysis on the aesthetic outcome was unable to perform due to limited data.		
Shahroom et al., 2020 [90]	Fluorosis	Systematic review RI showed a greater improvement in aesthetics in comparison to bleaching. RI with additional infiltration time and a combination of RI with bleaching is the best treatment options.		
Lopes et al., 2021 [91]	MIH	Umbrella review RI has been reported as useful. However, there is no strong evidence to support this technique.		
Bulanda et al., 2022 [92]	MIH	Systematic review RI significantly improved the aesthetics of MIH-affected teeth and improved the patients’ well-being. The depth of infiltration and the achievement of enamel hardness after such therapy are not precisely defined.		
Somani et al., 2022 [94]	MIH	Systematic review Resin infiltration techniques have shown some promise for MIH with white opacities There is insufficient evidence to support specific approaches for the management of MIH-affected incisors.		

RI—resin infiltration; WSL—white spot lesion; MIH—molar-incisor hypomineralisation; FV—fluoride varnish; CPP-ACP—casein-phosphopeptide amorphous calcium phosphate; H_2_O_2_—hydrogen peroxide.

**Table 4:** Summary of the main laboratory-based studies conducted to improve the current RI.

Incorporation of Remineralising Agents		
Study	Material	Summary of Findings
Sfalcin et al., 2017 [128]	Experimental resin consists of TEDGMA, BisEMA and one of the fillers: HAP, BAG, amorphous calcium phosphate or modified calcium silicate.	The addition of either HAP and BAG produced lower water sorption and solubility and significantly higher DC value, microhardness and modulus of elasticity as compared to the commercial RI.
Hashemian et al., 2021 [125]	Experimental resin consists of TEDGMA, PUA + either HAP or BAG fillers.	The experimental resin showed good penetration ability with an improved microhardness and modulus of elasticity. The addition of the fillers increased the water sorption and solubility of the resin. The addition of BAG releases higher calcium ions as compared to HAP-added RI.
Prodan et al., 2022 [127]	Experimental resin consists of different percentages of TEDGMA, HEMA, UDMA, Bis GMA with/without fluoride-releasing glass filler	The incorporation of the filler improves the DC and strength of the material and potentially inhibits demineralisation through the fluoride-releasing effect.
Dai et al., 2022 [129]	RI monomer + 10–30% of amorphous calcium phosphate	RI + 30% calcium phosphate is biocompatible and improves the hardness of the experimental resin, and it had the highest level of calcium and phosphate ion release.
Obeid et al., 2022 [130]	RI monomer + one of the following materials: nanofibers of PLA-SiO_2_ or nanofibers of PLA-SiO_2_-CaP	The addition of CaP increased the surface hardness and the DC value of the RI. Positive calcium and phosphate ion release were observed under the pH-cycling challenge for PLA-SiO_2_-CaP.
**Incorporation of antibacterial agents**		
Skucha-Nowak et al., 2016 [131]	Experimental RI consisting of TEDGMA, HEMA and a bioactive methacrylate monomer (PMMAn with build-in metronidazole)	The experimental resin with bacteriostatic properties managed to infiltrate the demineralised enamel lesion.
Inagaki et al., 2016 [132]	Experimental resin consists of TEDGMA and chlorhexidine diacetate salts of varying concentration	The incorporation of chlorhexidine diacetate salt did not affect the DC but improved the hardness of the experimental resin. The experimental resin inhibited the *S. mutans* effectively.
Flor-Riberio et al., 2019 [133]	Experimental resin consists of TEDGMA, BisGMA, diphenyliodonium salt and chitosan	The addition of iodonium salts improved the DC and microhardness of the experimental resin and inhibited the *S. mutans* and *L. acidophilus* biofilm.
Kielbassa et al., 2020 [97]	RI monomer + silver nanoparticles	The experimental resin penetrated the proximal dentinal lesion and occluded the pores similarly to the commercial RI. The presence of silver may enhance the antibacterial effects of the material.
Yu et al., 2020 [134]	RI monomer + quaternary ammonium monomer	The incorporation of quaternary ammonium monomer did not increase the cytotoxicity or change the physical properties of RI. The experimental resin reduced the amount of *S. mutans* biofilm and provided anti-demineralisation effects.
Collares et al., 2020 [140]	RI monomer + polyhexamethylene guanidine hydrochloride at 0.5 or 1% (by weight)	Complete eradication of *S. mutans* biofilm was achieved when in contact with the RI containing polyhexamethylene guanidine hydrochloride at 1% (by weight). Addition of the material did not affect the physical properties of RI.
Cuppini et al., 2021 [135]	RI monomer + microcapsule of ionic liquid BMI.NTf_2_	The addition of the filler produced similar tensile strength as the commercial RI. The filler increases the hydrophobicity and reduces the wettability of the resin, improving its hydrolytic stability in the oral environment. The filler also has antibiofilm effects.
Fisher et al., 2021 [136]	Experimental RI consisting of TEDGMA, HEMA and a bioactive methacrylate monomer (PMMAn with build-in metronidazole)	Both the experimental RI and the commercial RI penetrated not only the decalcified root cement but also reached the surface area of the root dentine.
**Self-etching RI**		
Wang et al., 2021 [142]	PAM and TEDGMA	The composition of 75% TEDGMA: 25% PAM showed similar penetration depth as compared to the control. The experimental resin also demonstrated acceptable viscosity, which allows the material to penetrate the lesion.
**Radiopaque RI**		
Moeinian 2018 [108]	RI monomer + one of these materials: SrHA, SrBG, barium methacrylate or tin methacrylate	The mixture of SrHA and SrBG with RI monomer resulted in a below-average radiopacity and increased the viscosity of the RI. The incorporation of barium and tin methacrylate produced a homogenous mixture with TEDGMA with low viscosity and contact angle values, but not clearly visible in the digital radiograph.
Pedreira et al., 2021 [143]	RI monomer + 45% zirconium oxide	The mixture of 45% zirconium oxide particles (by weight) with RI monomer produced radiopacity higher than enamel when viewed using a digital radiograph.
Nowak-Waschol et al., 2022 [144]	Yttrium trifluoride (YF_3_) incorporated in their experimental RI consisting of TEDGMA, HEMA and a bioactive methacrylate monomer (PMMAn with build-in metronidazole)	The penetration of the experimental resin into the carious root dentine was hampered by the inability of YF_3_ to penetrate deeper into the lesion due to its large particle size.
**Micro-filled RI**		
Askar et al., 2015 [145]	RI monomer + either one of these materials: Organic fillers of different particle sizes or glass filler at 0.7 µm	RI with 42 µm of organic filler displayed an indifferent penetration percentage as the commercial unfilled RI on an artificial caries model.
Askar et al., 2018 [146]	RI monomer + organic fillers at 42 µm	The micro-filled RI showed similar efficacy in penetrating the natural enamel carious lesion as the commercial unfilled resin but managed to fill the ICDAS 5 cavity

RI—resin infiltration; TEDGMA—triethylene glycol dimethacrylate; BisGMA—bisphenol A-glycidyl methacrylate; HEMA—2-hydroxyethyl methacrylate; BisEMA—ethoxylated bisphenol A dimethacrylate; UDMA-HAP—hydroxyapatite; BAG—bioactive glass; PUA—polyurethane acrylate oligomer; DC—degree of conversion; PLA-SiO_2_—polylactic acid-silicone oxide nanofibres; PLA-SiO_2_-CaP—calcium phosphate enhanced polylactic acid-silicone oxide nanofibers; PMMA—polymethyl methacrylate; BMI.NTf_2_-1-n-butyl-3-methylimidazolium bis(trifluoromethanesulfonyl)imide; PAM—phosphoric acid 2-hydroxyethyl methacrylate ester; SrHA—strontium hydroxyapatite; SrBG—strontium bioglass; ICDAS—International Caries Detection and Assessment System.

## Data Availability

Not applicable.
